# Delta-radiomics features for the prediction of patient outcomes in non–small cell lung cancer

**DOI:** 10.1038/s41598-017-00665-z

**Published:** 2017-04-03

**Authors:** Xenia Fave, Lifei Zhang, Jinzhong Yang, Dennis Mackin, Peter Balter, Daniel Gomez, David Followill, Aaron Kyle Jones, Francesco Stingo, Zhongxing Liao, Radhe Mohan, Laurence Court

**Affiliations:** 1 Department of Radiation Physics, The University of Texas MD Anderson Cancer Center, 1515 Holcombe Blvd, Houston, TX 77030 USA; 2 The University of Texas Graduate School of Biomedical Sciences at Houston, 6767 Bertner Ave, Houston, TX 77030 USA; 3 Department of Radiation Oncology, The University of Texas M.D. Anderson Cancer Center, 1515 Holcombe Blvd, Houston, TX 77030 USA; 4 Department of Imaging Physics, The University of Texas M.D. Anderson Cancer Center, 1515 Holcombe Blvd, Houston, TX 77030 USA; 5 Dipartimento Di Statistica, Informatica, Applicazioni, University of Florence, Viale Morgagni, 59, Florence, 50134 Italy

## Abstract

Radiomics is the use of quantitative imaging features extracted from medical images to characterize tumor pathology or heterogeneity. Features measured at pretreatment have successfully predicted patient outcomes in numerous cancer sites. This project was designed to determine whether radiomics features measured from non–small cell lung cancer (NSCLC) change during therapy and whether those features (delta-radiomics features) can improve prognostic models. Features were calculated from pretreatment and weekly intra-treatment computed tomography images for 107 patients with stage III NSCLC. Pretreatment images were used to determine feature-specific image preprocessing. Linear mixed-effects models were used to identify features that changed significantly with dose-fraction. Multivariate models were built for overall survival, distant metastases, and local recurrence using only clinical factors, clinical factors and pretreatment radiomics features, and clinical factors, pretreatment radiomics features, and delta-radiomics features. All of the radiomics features changed significantly during radiation therapy. For overall survival and distant metastases, pretreatment compactness improved the c-index. For local recurrence, pretreatment imaging features were not prognostic, while texture-strength measured at the end of treatment significantly stratified high- and low-risk patients. These results suggest radiomics features change due to radiation therapy and their values at the end of treatment may be indicators of tumor response.

## Introduction

Lung cancer is responsible for the largest number of cancer deaths in both men and women in the United States^1^. Over 85% of lung cancer cases are non-small cell lung cancers (NSCLC)^1, 2^. Predicting a particular NSCLC patient’s response to treatment, even compared to patients in the same disease stage group, is extremely difficult. A model that could effectively identify patients whose tumors are not responding to treatment would be beneficial and could be used to recommend patients for adjuvant chemotherapy or a radiation boost.

Radiomics is the extraction of quantitative imaging features from medical images. These quantitative values can be used to develop models for cancer diagnosis, patient prognosis, or relative tumor heterogeneity that can then guide clinical decisions^3, 4^. This process is similar to the current application of tumor stage or genetic information derived from tumor biopsy specimens for clinical decision making. Radiomics has the combined advantages of being highly patient-specific and non-invasive. Additionally, unlike biopsy specimens, radiomics allows for sampling the heterogeneity over the entire tumor. In recent years, numerous studies have examined the potential clinical utility of radiomics features calculated from computed tomography (CT) images of NSCLC. These studies have identified features that are linked to tumor histology^5, 6^, tumor stage^7^, patient overall survival^8–15^, and genetic mutations^16–18^.

Changes in radiomics features, called delta-radiomics features, have also been studied for their prognostic potential in cancer. Delta-radiomics features have been successful in predicting the response of colorectal cancer liver metastases^19^ and metastatic renal cell cancer^20^ to chemotherapies. Delta-radiomics features have also been used to identify patients with esophageal cancer who would develop radiation pneumonitis during treatment^21^. Delta-radiomics features calculated from PET images were shown to be predictive of overall survival for NSCLC patients^22^. To our knowledge, however, no published reports have investigated the possibility of using delta-radiomics features calculated from CT images for determining prognosis in NSCLC patients. Currently, NSCLC tumor response is analyzed with the Response Evaluation Criteria in Solid Tumors (RECIST) guidelines^23–25^. These guidelines depend on changes in tumor size to evaluate tumor response. Tumor size is widely known to be correlated with survival and probability for distant metastases in NSCLC. However, it does not reflect changes in tumor heterogeneity or genetic profiles, both of which may be more indicative of individual tumor biology. By sampling the entire tumor and analyzing changes in the spatial variations in intensity, delta-radiomics features may fill this gap and provide better patient-specific outcome predictions.

The main objective of this work was to determine whether therapy-induced changes in radiomics features, called delta-radiomics features, can improve models for predicting patient outcome when used in conjunction with clinical factors and radiomics features measured prior to treatment.

## Methods

### Patient data

For this study, we retrospectively reviewed the images and medical records for 137 NSCLC patients with a waiver of informed consent from the Institutional Review Board (IRB) at the University of Texas MD Anderson Cancer center. These patients were selected because they had been enrolled on an IRB approved clinical trial at the University of Texas MD Anderson Cancer Center where they were imaged weekly with a four-dimensional CT (4DCT) during their treatment^26^. Informed consent had been obtained from each study participant. All methods for the trial were performed in accordance with the University of Texas MD Anderson Cancer Center IRB guidelines and regulations and all experimental protocols were approved by the same IRB. Inclusion criteria for the trial were pathologically proven advanced NSCLC, Karnofsky performance status (KPS) ≥70 or ECOG score 0–1, forced expiratory volume ≥1 liter, and age between 18 and 85 years^26^. Exclusion criteria included small cell histology, prior radiotherapy to the planned radiation therapy fields, pregnancy, or oxygen dependence due to pre-existing lung disease^26^. The patients were treated with radiation therapy and concurrent chemotherapy. They had been randomized to receive treatment with either photons or protons to 66 or 74 Gy. Full trial details are available online^26^. In this retrospective analysis, treatment modality was not considered for classification purposes. For this retrospective analysis, the medical records of these patients were reviewed to determine their clinical factors: sex, age, smoking status, pack years, tumor histology, overall disease stage, T stage, N stage, KPS, and total prescribed radiation dose (Table 1). The primary endpoints for this retrospective analysis of the trial data were overall survival, freedom from distant metastases, and local-regional control.

**Table 1:** Clinical characteristics of the NSCLC patient population used for modeling in this analysis.

Clinical Factors	Number of Patients (n = 107)
Sex	
F	45
M	62
Age	
<65	45
>=65	62
T stage	
T1 or T2	49
T3 or T4	58
N stage	
N0 or N1	24
N2 or N3	83
Overall disease stage	
II	12
IIIa	44
IIIb	49
IV	2
Tumor histology	
Squamous cell carcinoma	46
Adenocarcinoma or other	61
Smoking status	
Current	34
Former	64
Never	9
Pack years	
0–24	20
25–49	37
50–74	28
75+	22
Karnofsky performance status	
90–100	52
70–80	55
Total radiation dose	
>70 Gy	72
<70 Gy	35

### Landmark analysis

Survival studies using measures of response that are calculated at multiple time points, such as the radiomics features measured at weekly intervals in this study, require a landmark time point to be used for calculating the time until the endpoint is reached^27, 28^. Otherwise a bias can be introduced by responders since they must have already survived to the time of treatment to be classified as responders^27, 28^. In this study, patients were classified as high or low risk using multivariate models that included clinical factors (recorded at the time of entrance to the study), pre-treatment radiomics features (measured from the treatment planning images), and delta-radiomics features (measured from the different weekly images through treatment). Because a variable number of days occurred for each patient between when they entered the trial and when their pre-treatment images were acquired and between their pre-treatment images and last weekly images, a landmark time point was required to measure survival. For this study, endpoints were defined from a landmark time point of 90 days from the day the patient was entered on the clinical trial until one of the endpoints of death, presence of distant metastases, or local-regional failure was met. Patients not reaching the endpoint were censored at their last follow-up date. The landmark point was calculated by determining the total number of days from entering the trial to end of treatment for each patient. The maximum interval (by which all measures of response had been determined) was 83 days, which was rounded to 90 days for simplicity. This ensures that the time until the endpoint is reached or the patient is censored is uniformly measured across all patients and not biased by the number of days they have already survived to reach the end of treatment.

### Imaging parameters

The pretreatment and weekly 4DCTs were acquired on either a GE Discovery ST or GE LightSpeed RT16 (GE Medical, Waukesha, WI) with a peak tube voltage of 120 kVp, tube current of 100 or 200 mA, and rotation times of 0.5 or 0.8 second. Axial images were reconstructed in a 512 × 512 matrix at an in-plane resolution of 0.98 mm and image thickness of 2.5 mm. These acquisition and reconstruction parameters are our institutional standards for CT imaging. For this study, the features were calculated from the end-of-exhale phase images for each scan. This phase was selected because it was considered the most stable and has been used in other radiomics studies^15, 29, 30^. The three-dimensional gross tumor volume contoured from the treatment plan was used as the region of interest (ROI) for feature extraction. The gross tumor volume contour from the treatment plan was deformably registered to each subsequent weekly 4DCT scan using clinical software developed in-house^31–33^. During this step, only the contour is deformed, the images themselves are not changed. All contours were visually inspected by the same person to ensure consistency and modified if necessary to remove any non-tumor regions. Furthermore, to ensure that normal lung and bone were excluded from the final ROI used for feature calculation, a thresholding step was also applied to each image with a lower threshold of −100 Hounsfield Units (HU) and upper threshold of 200 HU.

### Exclusion criteria

Patients were excluded from this dataset for any of the following reasons: (i) a small ROI volume (<5 cm^3^) (n = 18); (ii) imaging using a different protocol, such as breath hold instead of 4DCT (n = 9); or (iii) occurrence of an endpoint, e.g. death, before the landmark point used for calculating survival times (n = 3). After exclusion, 107 patients were included in the final data analysis.

### Feature extraction and selection

Feature calculation was performed using the IBEX software^34, 35^. This software allowed for customization of feature parameters and image preprocessing. Extracted features included shape features (n = 16), intensity histogram features (n = 11), co-occurrence matrix (COM) features (n = 22)^36, 37^, neighborhood gray-tone difference matrix (NGTDM) features (n = 5)^38^, and run-length matrix (RLM) features (n = 11)^39^. To determine the best parameters for the features, each feature was calculated four times: (1) once with no image preprocessing other than thresholding, (2) once with thresholding and smoothing using a Butterworth filter with an order of 2 and a cutoff of 125, (3) once with thresholding and an 8-bit depth resample, and (4) once with thresholding, Butterworth smoothing, and an 8-bit depth resample^40^. The Butterworth smoothing acts to remove Gaussian noise from the images, which may obscure the lower frequency biological variations that radiomics features are designed to measure. The 8-bit depth resample is used as an alternative to modifying the binning parameter for the histogram and radiomics matrices. Using 8-bit depth images results in a bin width of 16 HU and thus is more likely to reflect actual density changes in neighboring pixels than bins with a width of 1 HU, which largely reflect image noise.

**Figure 1:**
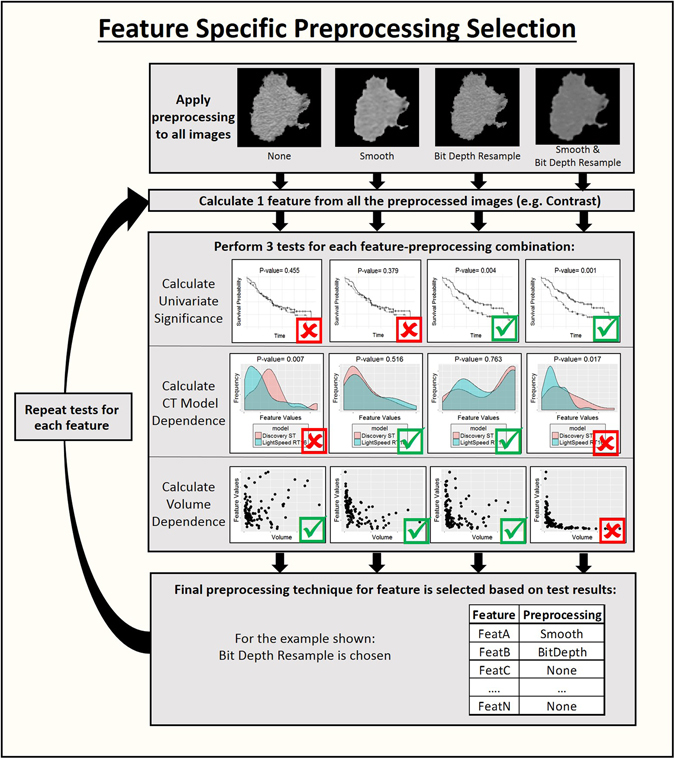
Workflow for the selection of feature specific image preprocessing. The images are all processed in four ways: no extra processing, smoothing with a Butterworth filter, resampling to an 8 bit depth, and both smoothing with a Butterworth filter and resampling with an 8 bit depth. Each feature is calculated from the four sets of processed images. Then the best processing is determined on a feature specific basis by evaluating the univariate significance, dependence on the CT model used to acquire the images, and volume dependence. Based on the results of these tests, one image preprocessing is selected for that feature. Then the process is repeated for the next feature. If no image preprocessing for a feature allows it to pass the tests, then the feature is removed entirely.

Optimal image pre-processing was determined on a feature-specific basis using the following steps which are also illustrated in Fig. 1. First a univariate Cox regression model for overall survival was fitted for each preprocessed version of each feature using only the pretreatment images for each patient. The significance of the feature in the model was calculated to determine whether a model built on only this feature was a better fit than the null model. This step identified radiomics features that were predictive and therefore might be useful for calculating delta-radiomics features. The correlation between each feature and the gross tumor volume was calculated using Spearman’s rank correlation coefficient. The feature values and gross tumor volumes were calculated from the pretreatment images for this step. Next a Wilcoxon rank sum test was performed for each feature and pre-processing combination to determine if the feature values were significantly different when images were acquired on the GE Discovery ST versus the GE Lightspeed RT16, as CT scanner model has been demonstrated to be an important factor in feature reproducibility^41^. A patient subset that had images available from the first week of treatment was used for this test because at this time point the patients were roughly split between the two CT scanners used in this study (37 patients imaged with the GE Discovery ST and 44 patients imaged with the GE Lightspeed RT16) and their tumors would not yet have shown any therapy-induced changes. For each feature, the pre-processed version that was significant in univariate analysis for survival (p-value < 0.10) and did not have a significant value (p-value > 0.05) for the Wilcoxon rank sum test between CT scanners was included in the final feature set. Features that never met these two criteria regardless of the image pre-processing used were excluded from the feature set. If a feature met both criteria for more than one image pre-processing type, the version of the feature that had the smallest correlation with volume was selected. A p-value of 0.10 was used as the threshold for significance in this pre-analysis because the p-values were used only for feature selection, not hypothesis testing, and thus the filtering need not be overly stringent. This choice was balanced against the need to remain conservative so that the feature dimensionality is decreased during this step. For the same reason, no multiplicity correction was used at this stage.

### Delta-radiomics features

Two tests were conducted to determine which of the optimized features changed during treatment and thus might be useful indicators of tumor response. First a linear mixed effects model with random intercepts for each patient was built for each feature in the form equation (1),

1 $${\rm{\Delta }}Feature\sim {\rm{\Delta }}Dose+({1}|{PatID})$$

Here, *ΔFeature* was the feature value measured from each weekly 4DCT, *ΔDose* was the total dose delivered to the tumor at that point in treatment, and *PatID* was a patient-specific identifier that allows the model to account for the fact that we had multiple, longitudinal measurements of each feature for each patient by assigning each patient their own intercept. The p-value of the log-likelihood ratio for each model was calculated. P-values were corrected for multiple comparisons using the Benjamini-Hochberg method^42^. If the corrected p-value was less than 0.05, the model was considered significant and indicated that the changes in the feature were significantly associated with the dose delivered to the tumor. For each feature with a significant p-value in this test, simplified measures of the overall change were calculated and defined as delta-radiomics features. The delta-radiomics features were defined as the relative net change, equation (2), the linear regression slope, and the value of the feature at the last week of treatment for each patient.

2 $${relativeNetChange}=({Featur}{{e}}_{{WeekFinal}}-{Featur}{{e}}_{{Week}1})/{Featur}{{e}}_{{Week}1}$$

Here, *Feature*_*WeekFinal*_ was the value of the feature at the end of treatment and *Feature*_*Week1*_ was the value at the first weekly 4DCT for each patient. A one-sample, two-tailed *t*-test was conducted for each of the delta-radiomics features to determine whether the overall changes for the group were significantly different from 0, and values were again corrected using the Benjamini-Hochberg method. Features that passed both the linear mixed effects and *t*-test analyses (corrected p-value < 0.05) were considered to significantly demonstrate radiation therapy-induced changes and were included as potential covariates in model building.

### Multivariate analysis

**Figure 2:**
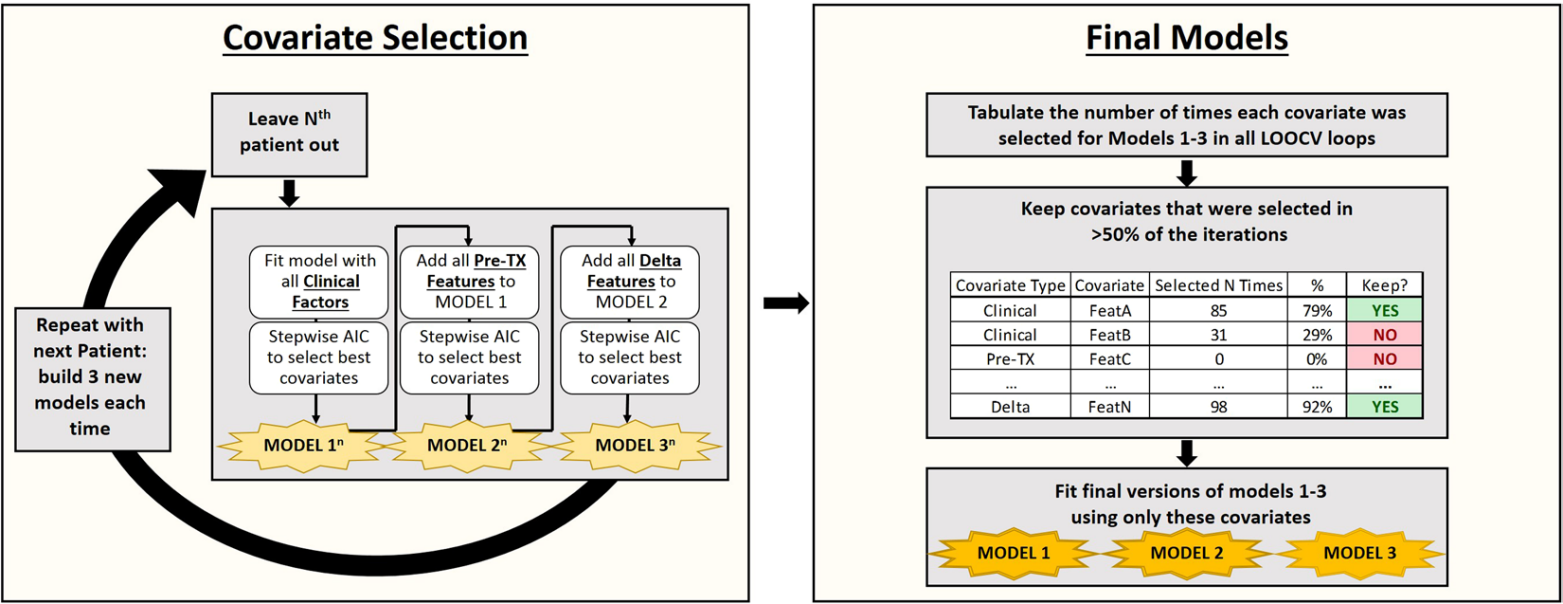
Workflow for building of multivariate models. A LOOCV loop is used to generate 3 models on each iteration: (1) Only clinical factors, (2) clinical factors and pre-treatment (TX) features, and (3) clinical factors, pre-TX features, and delta-radiomics features. After the three models have been built with each patient left out once, the number of times each covariate was selected is tabulated. Then those covariates that are selected in greater than 50% of the iterations are kept. These are then used to fit final versions of the 3 models.

Multivariate Cox regression models were built for each of the primary endpoints using leave-one-out cross validation (LOOCV) and Akaike Information Criterion (AIC) with the following procedure, which is also illustrated in Fig. 2. First, one patient was removed from the dataset and a Cox proportional hazards model was built using all of the clinical factors and the remaining patients. The covariates were reduced using stepwise AIC in both directions. Next, all of the pretreatment radiomics features were added to this model and stepwise AIC was repeated in both directions with forced nesting of the clinical covariates. Then, all of the delta-radiomics features were added to this model with forced nesting of the clinical and pretreatment radiomics covariates. The delta-radiomics versions of the features were identified by the suffixes “netPercentChange”, “Slope”, or “WeekLast”, while the pretreatment radiomics features are indicated by the suffix “Week0”. This process was repeated with each patient left out in turn so that at the end there were three models for each left-out patient: one with only clinical factors, one with clinical factors and pretreatment radiomics features, and one with clinical factors, pretreatment radiomics features, and delta-radiomics features. The total number of times each covariate was selected for the three models over all of the LOOCV iterations was calculated. Covariates that were selected in more than half of the iterations were retained and considered high-performing. Final versions of the three models using only these frequently selected covariates were then calculated and compared using the log-likelihood ratio to determine whether the radiomics and/or delta-radiomics features significantly (p-value < 0.05) improved the fit of the model to the data. If no feature was selected in more than half of the iterations for a particular model, then the null model or the nested model from the previous iteration was used.

To evaluate the prognostic potential of these features, a new LOOCV was performed. For this analysis, the three models were built on each iteration using only the high-performing clinical, radiomics, and delta-radiomics covariates from the original LOOCV. No covariate reduction was performed, but the coefficients were refit on each iteration. On each iteration of the loop, a prediction for the left-out patient was calculated using each of the three models. Because the patient was left out of the coefficient fitting process, predictions generated for the left out patient were unbiased. Once the loop was complete, and each patient had a prediction for each model, the Harrel concordance index^43^ (c-index) was calculated for each model. The c-index is analogous to the area under the curve but is designed for survival data instead of binary data. Values of the c-index can range from 0 to 1 with a value of 1 indicating perfect prediction and a value ≤ 0.5 indicating that a model performs no better or worse than a random guess. Thus the c-indices allowed for the comparison of the predictive accuracy of models that included radiomics and delta-radiomics features to models incorporating only clinical factors. Finally, patients were stratified as high or low risk based on whether their prediction was above or below the median prediction for each model. Kaplan-Meier curves were plotted using this patient stratification, and the log-rank test was used to determine whether the stratifications were significant (p-value < 0.05).

All statistical analyses were performed in R language^44^ using the survival^45^, lme4^46^, MASS^47^, and ggplot2^48^ analysis packages.

## Results

### Feature selection

The initial feature set had 49 texture features measured before treatment, with four different image preprocessing types and 16 shape features, for a total of 212 feature and preprocessing combinations. Of these, 75 were significant in univariate analysis (p < 0.10), and 123 were not significantly different between different CT scanners (p < 0.05). These results are shown in Supplementary Figures S1–S3. Using the feature selection process, this feature set was reduced to 31 features. Of these, 9 were calculated with no extra preprocessing, 15 were calculated with Butterworth smoothing, and 7 were calculated with Butterworth smoothing and 8-bit depth resampling (Supplementary Figure S4). At least one feature from every feature category was represented in this final feature set.

All 31 features had significant p-values for the log-likelihood ratio of their linear mixed effects model, with dose as the covariate and random intercepts for each patient, even after Benjamini-Hochberg correction for multiplicity. The net changes and slope in each feature were also significant in *t*-tests comparing their means to 0 after multiplicity correction for every feature. Thus a total of 31 features were available for feature selection in the multivariate model building.

### Multivariate analysis

For overall survival, 67 of the 107 patients reached the endpoint of death. The median survival time was 638 days. 50 patients had a distant metastases with a median time until reaching the endpoint or censoring of 311 days. 23 patients had a local recurrence with a median time until reaching the endpoint or censoring of 420 days. The final results of the multivariate analysis are summarized in Table 2 for all three outcomes and all three models. The number of times each clinical factor and radiomics feature was selected in the first LOOCV is tabulated in Supplementary Table S1 for each outcome.

**Table 2:** Final comparison of the three models for each outcome.

	Overall Survival	Distant Metastases	Local-regional Recurrence
C-index			
Model 1	0.597	0.539	NA
Model 2	0.672	0.632	NA
Model 3	0.675	NA	0.558
Log-likelihood ratio p-value			
Between model 1 and model 2	4.20 × 10^−5^***	4.87 × 10^−4^***	NA
Between model 1 and model 3	2.10 × 10^−5^***	NA	NA
Between model 2 and model 3	0.020*	NA	NA
Log-rank test p-value			
Model 1	5.27 × 10^−3^***	0.380	NA
Model 2	2.40 × 10^−6^***	1.56 × 10^−3^***	NA
Model 3	1.30 × 10^−5^***	NA	0.0269*

For the c-indices and log-rank test p-values, a value of NA indicates that no extra covariates were selected for this model and thus the value cannot be evaluated. Similarly, a value of NA for the log-likelihood ratio p-value implies that the two models being compared had the same covariates and thus the log-likelihood ratio cannot be computed. Model 1 is the model with only clinical factors. Model 2 is the model with clinical factors and pretreatment features. Model 3 is the model with clinical factors, pretreatment features, and delta-radiomics features. *Significant at p < 0.05; **Significant at p < 0.005; ***Significant at p < 0.001.

**Figure 3:**
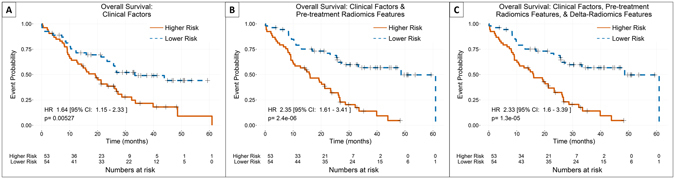
Comparison of Kaplan-Meier curves for overall survival using the three nested models with patients stratified by the median prediction value of each model. The stratification was significant for all three models. The addition of a pretreatment radiomics feature, compactness2, to clinical factors alone had a small improving effect on the stratification, while the further addition of delta-radiomics features had almost no impact on the stratification.

Adding the single selected pretreatment feature compactness2 increased the c-index from 0.597 to 0.672 for overall survival. The log-likelihood ratio between these two models was significant. However, further addition of delta-radiomics features made a negligible difference to the c-index and did not substantially affect the patient stratification by the Kaplan-Meier curves (Fig. 3). The log-likelihood ratio between model 2 (with clinical factors and pretreatment radiomics features) and model 3 (with clinical factors, pretreatment radiomics features, and delta-radiomics features) was significant, indicating an improved fit. The clinical factors included in the final model were T stage, patient sex, tumor histology, and total radiation dose. The pretreatment feature that was included was compactness2 from the shape category. The delta-radiomics features that were included were the slopes in grey-level non-uniformity from the RLM and texture strength calculated from the NGTDM.

**Figure 4:**
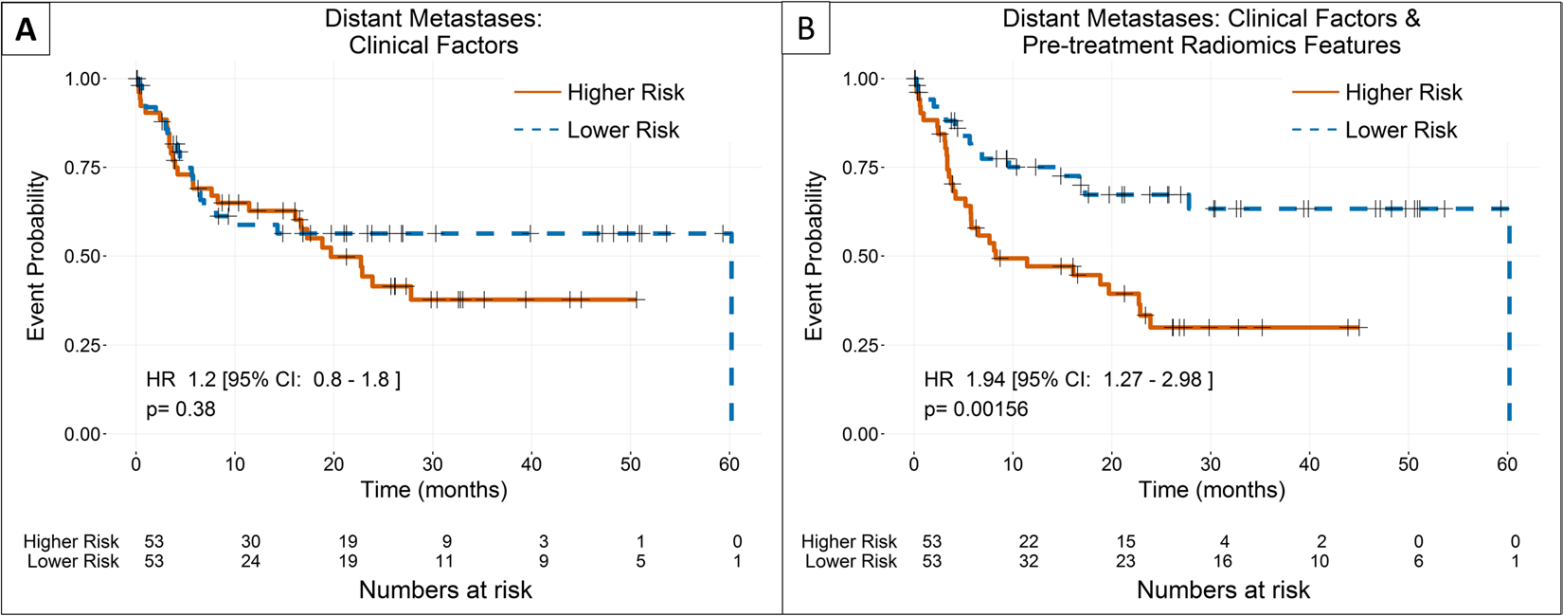
Comparison of Kaplan-Meier curves for distant metastases using the two nested models with patients stratified by the median predicted value of each model. No delta-radiomics features were selected in the model-building process for distant metastases, so only two models were available. For distant metastases, the addition of a pretreatment radiomics feature, compactness2, significantly improved the stratification of the patients.

For distant metastases, no delta-radiomics features were included in the final model. The final clinical factors included in the model were tumor T stage, overall disease stage, and patient age, sex, and smoking status. Adding a pretreatment feature, compactness2 from the shape category, did result in an increase in the c-index from 0.539 to 0.632. The log-likelihood ratio between model 1 (clinical factors only) and model 2 (clinical factors and pretreatment radiomics features) was highly significant. Furthermore, patient stratification was significant when the pretreatment radiomics feature was added, while it was not significant for the purely clinical model (Fig. 4).

**Figure 5:**
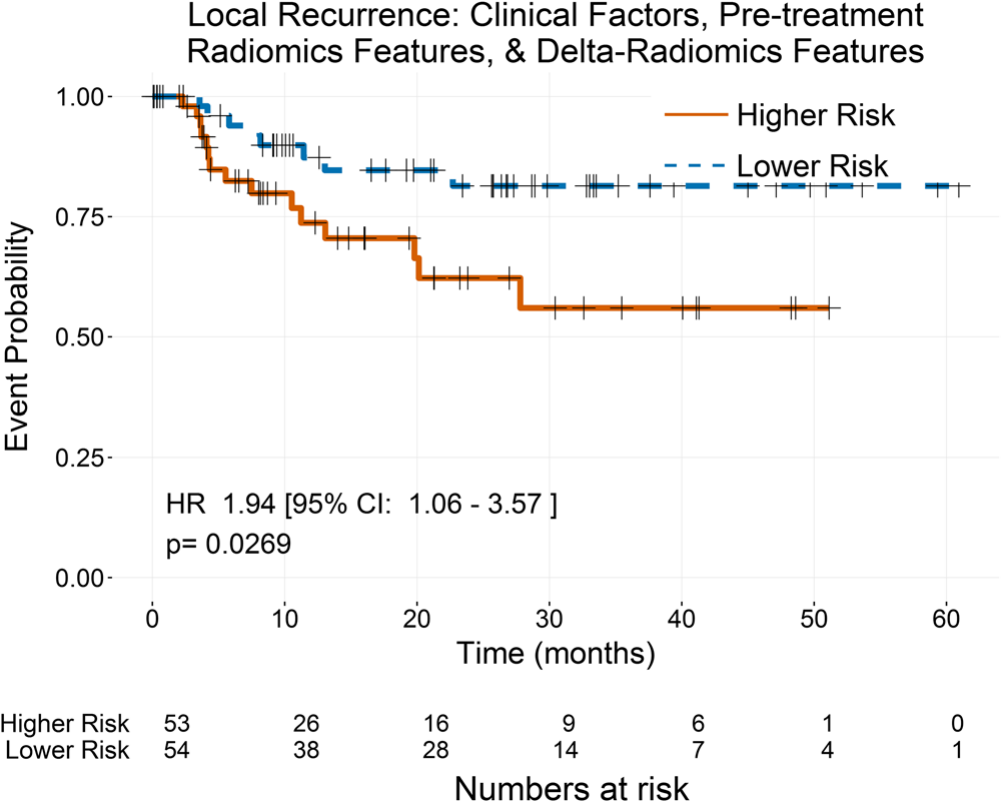
Kaplan-Meier curves for local-regional recurrence using the delta-radiomics model with patients stratified by the median predicted value from the model. The only covariate selected in the model building process for local recurrence, was the delta-radiomics feature texture strength measured at the end of treatment. When patients were stratified by the median of their predicted values, the resulting Kaplan-Meier curves were significantly stratified.

For local-regional recurrence, no clinical factors or pretreatment radiomics features were selected in more than half of the LOOCV iterations. As a result, none were considered high-performing or were available for use in the final models for local recurrence. However, the delta-radiomics feature texture strength from the NGTDM measured at the end of treatment was selected in a majority of the LOOCV iterations. As a result, only the model including delta-radiomics features was built. This univariate model resulted in a low value for the c-index (0.558) but a statistically significant stratification of the patients (p-value = 0.0269; Fig. 5). In lieu of calculating the log-likelihood ratio between this model and model 1 (clinical features only) or model 3 (clinical, pretreatment radiomics, and delta-radiomics factors), the log-likelihood ratio between this model and the null model was calculated (p-value = 0.0725).

## Discussion

While the inclusion of delta-radiomics features had a statistically significant impact on the overall likelihood of a model for overall survival compared to a model with only clinical and pretreatment radiomics features, the impact on the model’s prognostic abilities was generally negligible. For distant metastases, no delta-radiomics features were selected in the final round of model building. This suggests that delta-radiomics features do not offer substantially new prognostic information for these outcomes though they were still prognostic for overall survival. The same pretreatment radiomics feature, compactness2, was selected for both the overall survival and time to distant metastases models and improved their prognostic potential. For both overall survival and distant metastases, the coefficient for this feature was positive, meaning a patient had a higher predicted risk of experiencing the outcome if the value for compactness2 from their ROI was relatively large. This feature was related to the volume and shape of the tumor ROI, i.e. how spiculated it may appear. The feature values were also affected by the tumor location, since a tumor attached to the chest wall was contoured with at least one smooth side compared to a tumor surrounded by lung which ranged anywhere between fully smooth or fully spiculated. Compactness 2 was also found to be predictive in a radiomics study by Aerts *et al*. where it was included as part of a four feature radiomics signature^11^. This study is unique in that it demonstrated that compactness 2 added significant new information to a variety of clinical factors already routinely obtained, as opposed to only TNM staging and tumor volume. In this study, the clinical model was built first and then radiomics features were added to it rather than building a purely radiomics model and assessing its capabilities. This is important because the introduction of radiomics features into a routine clinical workflow is unlikely to be accepted unless models built using radiomics features outperform models built using only routinely acquired clinical factors.

Interestingly, in the models for local-regional recurrence, the only covariate that was predictive for outcomes was a radiomics feature, texture strength from the NGTDM, measured from images acquired during the last week of treatment. This feature was designed to quantify whether an image has clear, perceivable characteristics that can be considered as texture and the overall strength of that signal^38^. Further work is needed to identify what this feature may represent in the context of NSCLC tumor analysis. This result may be evidence that, although it is not possible to predict local-regional recurrence prior to treatment, the state of the tumor at the end of the treatment can be assessed using radiomics.

One possible cause of the poor selection of delta-radiomics features in the models may be due to the initial feature preselection process. The full feature set was first reduced to features whose pretreatment values were at least prognostic in univariate models for overall survival. It is possible that the results would differ if this requirement was changed to instead select for delta-radiomics features that are significant in univariate models. The original requirement was chosen for two reasons: first, because several publications have shown that pretreatment radiomics features have informative value and thus changes in the features that are already prognostic may reflect actual biological changes in the tumor, and second, if model building was limited to delta-radiomics features that were significant in univariate analyses the results could be biased and overly optimistic.

One limitation of this study was the lack of a dataset for independent model validation due to the fact that patients are not routinely imaged weekly during their treatment. This limitation was mitigated by using cross validation, which has been shown to be an effective method for creating unbiased patient-specific predictions^49, 50^. Another limitation of this study was that the median predicted value was used as the cut-off point for high- and low-risk patients. This is not an optimized approach, and it is very likely that a different model-specific value would yield different results. However, testing multiple cutoffs to find the best one without an independent validation dataset to test it in has been repeatedly shown to yield overly optimistic results^51–53^. By using the median, this source of bias is avoided and the conclusions remained conservative. Lastly, because the images used in this analysis were non-contrast CT images, vessels passing through the lesion could not be segmented from the contour. Thus the contours for the tumor ROIs may contain vasculature along with the solid tumor component we are interested in. The inclusion of vasculature in the tumor ROIs may affect the radiomics features and the calculated tumor volume.

Radiomics is in some ways fundamentally limited because the features are not inherently descriptive. This is in contrast to clinical covariates which, when selected in prognostic models, lend themselves to hypotheses, e.g., age is likely to affect survival because a younger person is statistically likelier to live longer than an older person. For radiomics features, this type of reasoning is difficult and instead new studies must be undertaken to correlate feature values with biological characteristics such as genetic mutations. Radiomics features also suffer from lack of robustness, as they have been demonstrated to vary with imaging equipment, ROI contouring, and imaging parameters. Thus the implementation of radiomics features in a clinical setting would require substantial effort to standardize both imaging and measurement parameters. This study identified two features, compactness2 and texture strength, which may be of clinical significance. The first step in determining their robustness will be to examine the impact of segmentation on both features’ values and prognostic potentials. This is especially critical for compactness2 since it is a shape based feature and thus could be substantially impacted by segmentation. In conclusion, this study found evidence that radiomics features change during the course of radiation therapy for NSCLC. However, these changes in features did not significantly outperform features measured before treatment in multivariate models for overall survival and distant metastases. Thus it may be more important to focus efforts on improving the standardization of features measured before treatment and identifying a biological or molecular explanation for their predictive values. One radiomics feature measured at the end of treatment did outperform both clinical factors and pretreatment radiomics features for prediction of local-regional recurrence. This feature, texture-strength, could become an indicator for tumor response since it was only prognostic when measured at the end of treatment. Despite the fact that this study did not find strong evidence supporting the prognostic potential of delta-radiomics features, the results of this study are important because the potential of tracking radiomics features throughout treatment for NSCLC was investigated. Furthermore, while other studies have used delta-radiomics features for other treatment sites or for normal tissue toxicity, they have used only the relative net change in their models^21, 22, 54^. This study included both the slope of a linear regression for the features of each patient, which may be less susceptible to noise than the relative net change, and the feature values at the end of treatment, which may reflect tumor response.

## Electronic supplementary material

Supplementary Data
