# Supplementary material for: Delta-radiomics features for the prediction of patient outcomes in non–small cell lung cancer
Source: Sci Rep. 2017 Apr 3;7:588. doi: 10.1038/s41598-017-00665-z (PMC5428827; doi:10.1038/s41598-017-00665-z)
Supplement: Supplementary file 1 — Supplementary Data [file 41598_2017_665_MOESM1_ESM.pdf]

SUPPLEMENTARY MATERIAL

Delta-radiomics features for the prediction of patient outcomes in non–small cell lung cancer

Xenia Fave*[1, 2], Lifei Zhang[1], Jinzhong Yang[1], Dennis Mackin[1], Peter Balter[1], Daniel Gomez[3], David Followill[1], Kyle Jones[4], Francesco Stingo[5], Zhongxing Liao[3], Radhe Mohan[1], and Laurence Court[1, 2].

[1]Department of Radiation Physics, The University of Texas MD Anderson Cancer Center 1515 Holcombe Blvd, Houston, TX 77030.

[2]The University of Texas Graduate School of Biomedical Sciences at Houston, 6767 Bertner Ave, Houston, TX 77030.

[3]Department of Radiation Oncology, The University of Texas M.D. Anderson Cancer Center, 1515 Holcombe Blvd, Houston, TX 77030.

[4]Department of Imaging Physics, The University of Texas M.D. Anderson Cancer Center, 1515 Holcombe Blvd, Houston, TX 77030.

[5]Department of Biostatistics, The University of Texas M.D. Anderson Cancer Center, 1515 Holcombe Blvd, Houston, TX 77030.

*****Xenia Fave

UTMD Anderson Cancer Center

Department of Radiation Physics-Unit 1420

1400 Pressler St, Houston, TX 77030

Office Phone: 713-745-6751

Email: xjfave@mdanderson.org

Table S1: Radiomics features analyzed in this study and their abbreviations.

Feature Category	Feature	Feature Abbreviation
Histogram	Variance	HISTvar
	Uniformity	HISTunif
	Standard deviation	HISTstd
	Skewness	HISTskew
	Minimum	HISTmin
	Median	HISTmed
	Mean	HISTmean
	Maximum	HISTmax
	Kurtosis	HISTkurt
	Entropy	HISTentropy
	Energy	HISTenergy
Run-Length Matrix	Short run low gray-level emphasis	RLMsrlgle
	Short run emphasis	RLMsre
	Run percentage	RLMrunperc
	Run-length non-uniformity	RLMrlnu
	Long run low gray level emphasis	RLMlrlgle
	Long run high gray level emphasis	RLMlrhgle
	Long run emphasis	RLMlre
	Low gray-level run emphasis	RLMlglre
	High gray-level run emphasis	RLMhglre
	Gray-level non-uniformity	RLMglnu
Neighborhood Gray-tone Difference Matrix	Texture Strength	NDMtexstr
	Contrast	NDMcontrast
	Complexity	NDMcomp
	Coarseness	NDMcoarse
	Busyness	NDMbusy

Co-occurrence Matrix	Variance	COMvar
	Sum Variance	COMsumvar
	Sum Entropy	COMsument
	Sum Average	COMsumavg
	Max Probability	COMmaxprob
	Inverse Variance	COMinvvar
	Inverse Difference Norm	COMinvdifn
	Inverse Difference Moment Norm	COMinvdifmn
	Information Measure Correlation	COMinfomc
	Information Measure Correlation 2	COMinfomc2
	Homogeneity	COMhomog
	Homogeneity 2	COMhomog2
	Entropy	COMentropy
	Energy	COMenergy
	Dissimilarity	COMdissim
	Difference Entropy	COMdiffent
	Correlation	COMcorrel
	Contrast	COMcontrast
	Cluster Tendency	COMclustend
	Cluster Shade	COMclusshade
	Autocorrelation	COMautocorrel

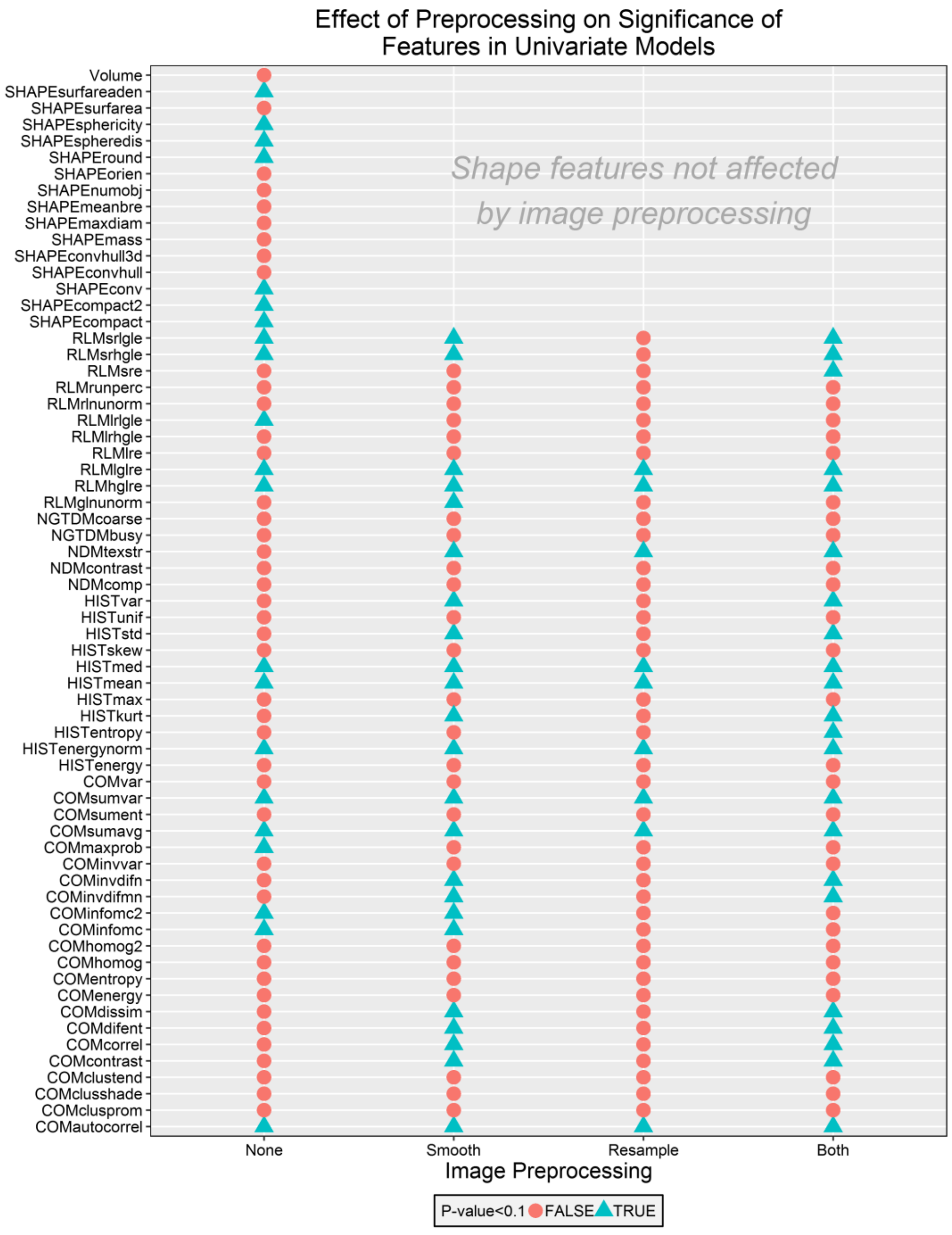

Figure S1: Impact of image preprocessing on the univariate significance of radiomics features. This figure plots each radiomics feature versus the image preprocessing styles used to calculate it. Blue triangles signify that the Feature-Preprocessing combination resulted in a significant fit for a univariate Cox regression using only the features measured at pretreatment (p-value <0.05). Red circles indicate that the univariate fit was not significant and thus that the feature should not be measured with that preprocessing style. Note that the shape features do not change with image preprocessing and so were calculated only with the basic thresholding step. None means that the feature was calculated with only a simple thresholding step, Smooth that the feature was calculated with Butterworth smoothing and

thresholding, Resample that the feature was calculated with thresholding and 8-bit depth resampling, and Both that the feature was calculated with Butterworth smoothing, thresholding, and 8-bit depth resampling.

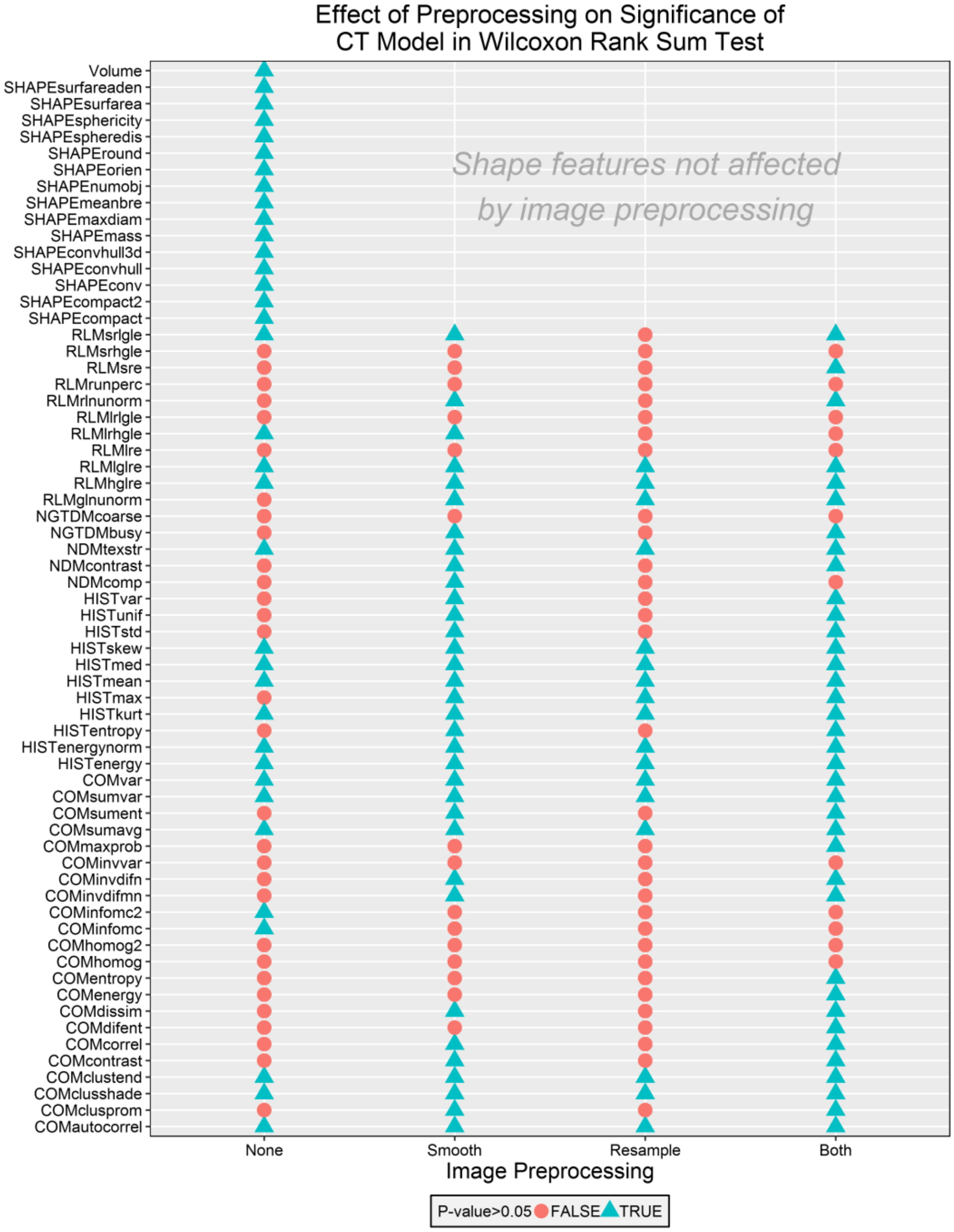

Figure S2: Impact of image preprocessing on the significance of CT Model in a Wilcoxon rank sum test for each radiomics feature. This figure plots each radiomics feature versus the image preprocessing styles used to calculate it. Blue triangles signify that the Feature-Preprocessing combination did not have a significant p-value (i.e., <0.05) in the Wilcoxon rank sum test for the impact of the CT Model (GE Lightspeed RT16 vs GE Discovery ST). Red circles indicate that the p-value was significant and thus that the feature calculated with that preprocessing style was significantly affected by the CT scanner model with which the images were acquired. Note that the shape features do not change with image

preprocessing and so were calculated only with the basic thresholding step. None means that the feature was calculated with only a simple thresholding step, Smooth that the feature was calculated with Butterworth smoothing and thresholding, Resample that the feature was calculated with thresholding and 8-bit depth resampling, and Both that the feature was calculated with Butterworth smoothing, thresholding, and 8-bit depth resampling.

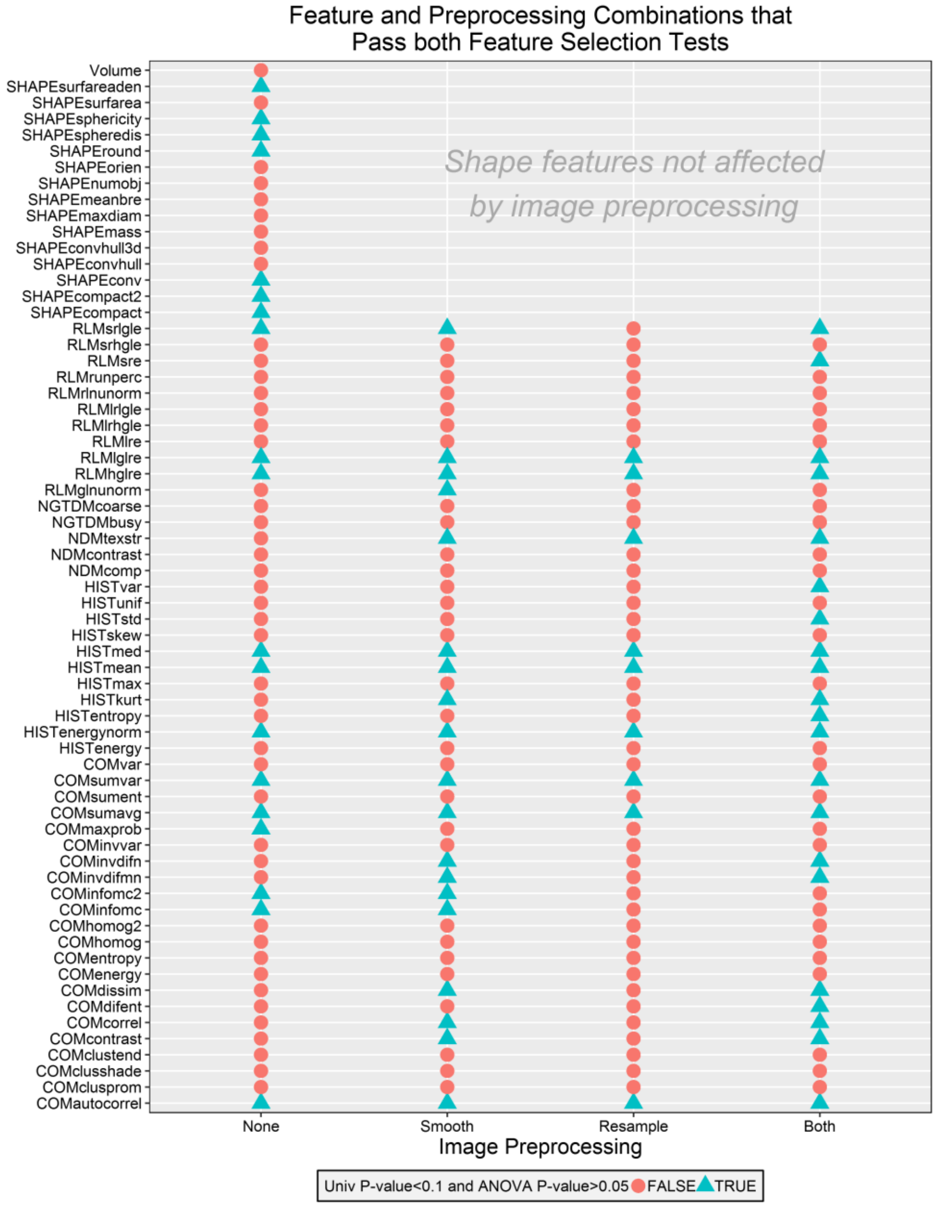

Figure S3: Impact of image preprocessing on the univariate significance and significance of the CT Model in a Wilcoxon rank sum test for each radiomics feature. This figure plots each radiomics feature versus the image preprocessing styles used to calculate it. Blue triangles signify that the Feature-Preprocessing combination passed both tests (had a significant p-value [<0.1] in the univariate analysis and did not have a significant p-value [<0.05] in the Wilcoxon rank sum test analyzing the impact of CT scanner model). Red circles indicate that the feature failed at least one test. Note that the shape features do not change with image preprocessing and so were calculated only with the basic thresholding step. None means that the feature was calculated with only a simple thresholding step, Smooth that the feature was calculated with Butterworth smoothing and thresholding, Resample that the feature was calculated

with thresholding and 8-bit depth resampling, and Both that the feature was calculated with Butterworth smoothing, thresholding, and 8-bit depth resampling.

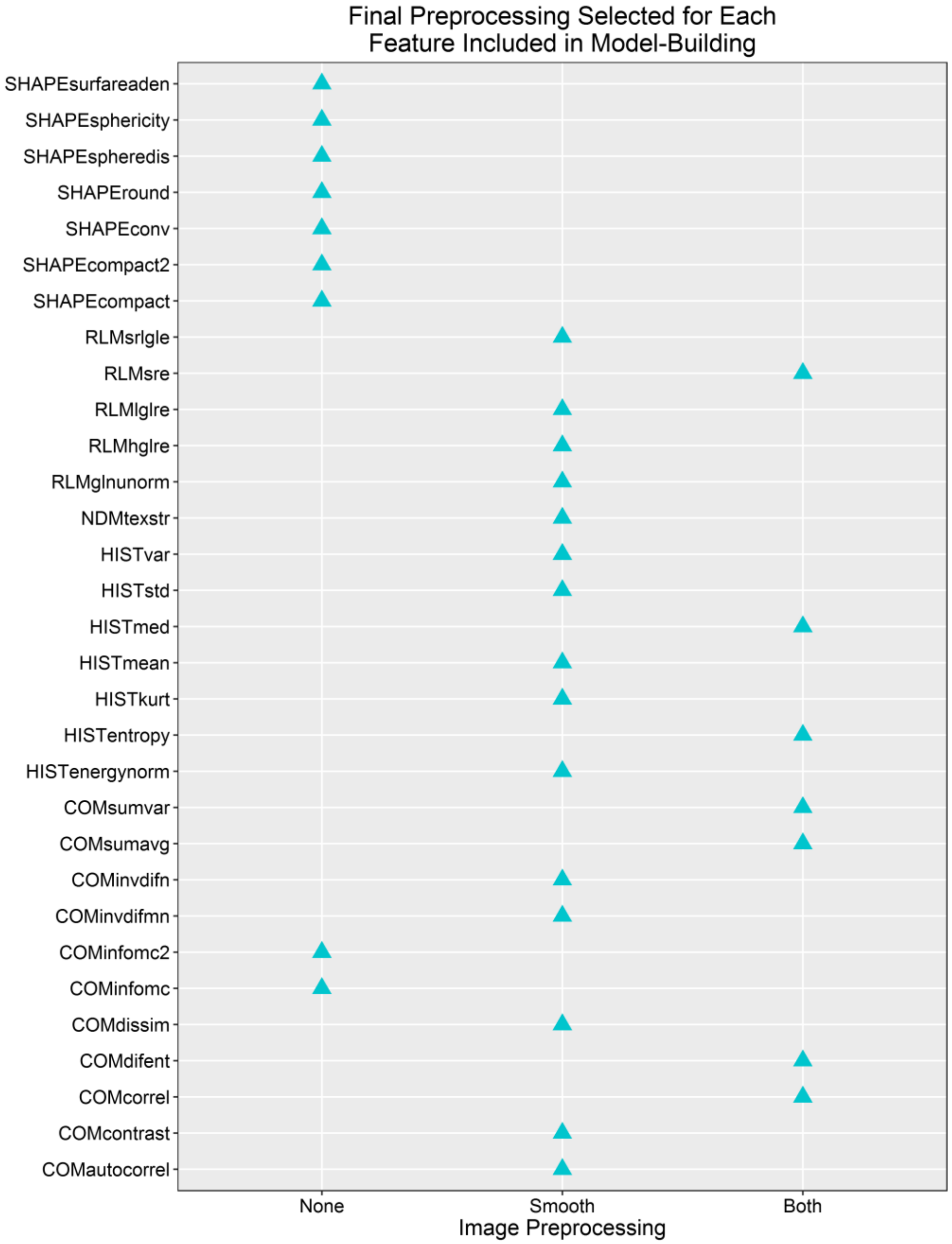

Figure S4: Final image preprocessing that was selected for each radiomics feature used in the analysis. This figure shows the final set of features that remained after our entire feature selection process along with the feature-specific image preprocessing chosen for each. None means that the feature was calculated with only a simple thresholding step, Smooth that the feature was calculated with Butterworth smoothing and thresholding, Resample that the feature was calculated with thresholding and 8-bit depth resampling, and Both that the feature was calculated with Butterworth smoothing, thresholding, and 8-bit depth resampling.

Table S2: Clinical factors and radiomics features selected in leave-one-out cross validation

Model	Feature Type	Covariates	No. of times selected in LOOCV	Included in final model	Model Coefficient
**Overall Survival**	Clinical Factors	T stage (T stage 3-4)	107	Yes	-0.933
		Sex (male)	107	Yes	0.866
		Histology (SCC)	107	Yes	0.772
		Total radiation dose (>70 Gy)	107	Yes	-0.652
		KPS	1	No	
		Pack years	1	No	
	WK0 Features	SHAPEcompact2.Week0	107	Yes	0.546
	Delta Features	RLMglnu.Slope	96	Yes	-0.240
		NGTDMtexstr.Slope	67	Yes	-0.330
		HISTkurt.netPercentChange	26	No	
		NGTDMtexstr.WeekLast	13	No	
		HistKurt.WeekLast	3	No	
**Distant Metastases**	Clinical Factors	Age (≥65 years)	93	Yes	-0.467
		Sex (male)	86	Yes	0.492
		Overall stage (≥IIIB)	61	Yes	0.552
		T stage (T stage 3-4)	59	Yes	-0.490
		Smoking status (former)	58	Yes	-0.062
		Smoking status (current)	58	Yes	0.060
		Total radiation dose	46	No	
		KPS	11	No	
	WK0 Features	SHAPEcompact2.Week0	105	Yes	0.585
		COMsumvar.Week0	13	No	
		SHAPEcompact.Week0	1	No	
		HISTkurt.Week0	1	No	
		COMautocorrel.Week0	1	No	
	Delta Features	COMcorrel.netPercentChange	7	No	
		COMinfomc2.WeekLast	6	No	
		COMinvdifmn.WeekLast	2	No	
		COMinvdifmn.Slope	1	No	

		COMinvdifmn.netPercentChange	1	No	
**Local Recurrence**	Clinical Factors	Smoking status (former)	19	No	
		Smoking status (current)	19	No	
		KPS	15	No	
		Pack years	3	No	
	WK0 Features	NGTDMtexstrength.Week0	17	No	
		ShapeSphereDisprop.Week0	1	No	
	Delta Features	NGTDMtexstrength.WeekLast	88	Yes	-0.517
		ShapeRoundeness.netpercentChange	13	No	
		HistKurt.WeekLast	1	No	

The number of times each clinical factor and radiomics feature was selected in the first leave-one-out cross validation are tabulated for each outcome. Features included in the final model have their model coefficients included in the table. Abbreviations: LOOCV, leave one out cross validation; SCC, squamous cell carcinoma; KPS, Karnofsky Performance Status; WK0, week 0 (i.e., pretreatment)

Table S3: Reproduction of the feature-parameters sheet produced by the radiomics analysis software IBEX for the calculation of the final set of radiomics features used in this study.

**FeatureItem-1**					
**Category**	**Parameters**	**Feature**	**Parameters**	**Preprocess**	**Parameters**
Shape		Compactness1		Threshold_Image_Mask	ThresholdLow=900; ThresholdHigh=1200; ErosionDist=0;
		Compactness2			
		Convex			
		Roundness			
		SphericalDisproportion			
		Sphericity			
		SurfaceAreaDensity			
**FeatureItem-2**					
**Category**	**Parameters**	**Feature**	**Parameters**	**Preprocess**	**Parameters**
GrayLevelCooccurenceMatrix25	Direction=0 45 90 135; AdaptLimitLevel=1; GrayLimits=0 4096; NumLevels=4096; Offset=1; Symmetric=0;	InformationMeasureCorr1		Threshold_Image_Mask	ThresholdLow=900; ThresholdHigh=1200; ErosionDist=0;
		InformationMeasureCorr2			
**FeatureItem-3**					
**Category**	**Parameters**	**Feature**	**Parameters**	**Preprocess**	**Parameters**
GrayLevelRunLengthMatrix25	Direction=0 90; GrayLimits=1 4096; NumLevels=4096;	GrayLevelNonuniformity		Butterworth_Smooth	cutoff=125; order=2; x_padded_size=512; y_padded_size=512; draw_before_after=0; images_folder=;
		HighGrayLevelRunEmpha		Threshold_Image_Mask	ThresholdLow=900; ThresholdHigh=1200; ErosionDist=0;
		LowGrayLevelRunEmpha			
		ShortRunLowGrayLevelEmpha			
**FeatureItem-4**					
**Category**	**Parameters**	**Feature**	**Parameters**	**Preprocess**	**Parameters**
NeighborIntensityDifference25	NHood=5; NHoodSym=1; IncludeEdge=0; AdaptLimitLevel=1; RangeMin=0; RangeMax=4096; NBins=4096;	TextureStrength		Butterworth_Smooth	cutoff=125; order=2; x_padded_size=512; y_padded_size=512; draw_before_after=0; images_folder=;
				Threshold_Image_Mask	ThresholdLow=900; ThresholdHigh=1200; ErosionDist=0;
**FeatureItem-5**					
**Category**	**Parameters**	**Feature**	**Parameters**	**Preprocess**	**Parameters**
IntensityDirect	ThresholdLow=1; ThresholdHigh=8000; ErosionDist=0; OnlyUseMaxSlice=0;	EnergyNorm		Butterworth_Smooth	cutoff=125; order=2; x_padded_size=512; y_padded_size=512; draw_before_after=0; images_folder=;
		GlobalMean		Threshold_Image_Mask	ThresholdLow=900; ThresholdHigh=1200; ErosionDist=0;
		GlobalStd			
		Kurtosis			
		Variance			
**FeatureItem-6**					
**Category**	**Parameters**	**Feature**	**Parameters**	**Preprocess**	**Parameters**
GrayLevelCooccurenceMatrix25	Direction=0 45 90 135; AdaptLimitLevel=1; GrayLimits=0 4096; NumLevels=4096; Offset=1; Symmetric=0;	AutoCorrelation		Butterworth_Smooth	cutoff=125; order=2; x_padded_size=512; y_padded_size=512; draw_before_after=0; images_folder=;
		Contrast		Threshold_Image_Mask	ThresholdLow=900; ThresholdHigh=1200; ErosionDist=0;
		Dissimilarity			
		InverseDiffMomentNorm			
		InverseDiffNorm			
**FeatureItem-7**					
**Category**	**Parameters**	**Feature**	**Parameters**	**Preprocess**	**Parameters**
GrayLevelRunLengthMatrix25	Direction=0 90; GrayLimits=1 256; NumLevels=256;	ShortRunEmphasis		Butterworth_Smooth	cutoff=125; order=2; x_padded_size=512; y_padded_size=512; draw_before_after=0; images_folder=;
				Threshold_Image_Mask	ThresholdLow=900; ThresholdHigh=1200; ErosionDist=0;
				BitDepthRescale_Range	RangeMin=0; RangeMax=4096; RangeFix=1; BitDepth=8;
**FeatureItem-8**					
**Category**	**Parameters**	**Feature**	**Parameters**	**Preprocess**	**Parameters**
IntensityDirect	ThresholdLow=1; ThresholdHigh=8000; ErosionDist=0; OnlyUseMaxSlice=0;	GlobalEntropy	NBins=256; RangeMin=0; RangeMax=4096; RangeFix=0;	Butterworth_Smooth	cutoff=125; order=2; x_padded_size=512; y_padded_size=512; draw_before_after=0; images_folder=;
		GlobalMedian		Threshold_Image_Mask	ThresholdLow=900; ThresholdHigh=1200; ErosionDist=0;
				BitDepthRescale_Range	RangeMin=0; RangeMax=4096; RangeFix=1; BitDepth=8;
**FeatureItem-9**					
**Category**	**Parameters**	**Feature**	**Parameters**	**Preprocess**	**Parameters**
GrayLevelCooccurenceMatrix25	Direction=0 45 90 135; AdaptLimitLevel=1; GrayLimits=1 256; NumLevels=256; Offset=1; Symmetric=0;	Correlation		Butterworth_Smooth	cutoff=125; order=2; x_padded_size=512; y_padded_size=512; draw_before_after=0; images_folder=;
		DifferenceEntropy		Threshold_Image_Mask	ThresholdLow=900; ThresholdHigh=1200; ErosionDist=0;
		SumAverage		BitDepthRescale_Range	RangeMin=0; RangeMax=4096; RangeFix=1; BitDepth=8;
		SumVariance			

Note that the threshold values in this table represent the values used for the patient ROIs and are represented in pinnacle units which have an offset of +1000 from Hounsfield units.
